# The metabolic signature of blood lipids: a causal inference study using twins

**DOI:** 10.1016/j.jlr.2024.100625

**Published:** 2024-09-19

**Authors:** Yutong Wang, Shunkai Liu, Weihua Cao, Jun Lv, Canqing Yu, Tao Huang, Dianjianyi Sun, Chunxiao Liao, Yuanjie Pang, Zengchang Pang, Min Yu, Hua Wang, Xianping Wu, Yu Liu, Wenjing Gao, Liming Li

**Affiliations:** 1 Department of Epidemiology and Biostatistics, School of Public Health, Peking University, Beijing, China; 2 Key Laboratory of Epidemiology of Major Diseases (Peking University), Ministry of Education, Beijing, China; 3 Peking University Center for Public Health and Epidemic Preparedness & Response, Beijing, China; 4 Qingdao Center for Disease Control and Prevention, Qingdao, China; 5 Zhejiang Center for Disease Control and Prevention, Hangzhou, China; 6 Jiangsu Center for Disease Control and Prevention, Nanjing, China; 7 Sichuan Center for Disease Control and Prevention, Chengdu, China; 8 Heilongjiang Center for Disease Control and Prevention, Harbin, China

**Keywords:** twin study, metabolomics, blood lipids, causal inference, cardiometabolic traits

## Abstract

Dyslipidemia is one of the cardiometabolic risk factors that influences mortality globally. Unraveling the causality between blood lipids and metabolites and the complex networks connecting lipids, metabolites, and other cardiometabolic traits can help to more accurately reflect the body’s metabolic disorders and even cardiometabolic diseases. We conducted targeted metabolomics of 248 metabolites in 437 twins from the Chinese National Twin Registry. Inference about Causation through Examination of FAmiliaL CONfounding (ICE FALCON) analysis was used for causal inference between metabolites and lipid parameters. Bidirectional mediation analysis was performed to explore the linkages between blood lipids, metabolites, and other seven cardiometabolic traits. We identified 44, 1, and 31 metabolites associated with triglyceride (TG), total cholesterol (TC), and high-density lipoprotein-cholesterol (HDL-C), most of which were gut microbiota-derived metabolites. There were 9, 1, and 14 metabolites that showed novel associations with TG, TC, and HDL-C, respectively. ICE FALCON analysis found that TG and HDL-C may have a predicted causal effect on 23 and six metabolites, respectively, and one metabolite may have a predicted causal effect on TG. Mediation analysis discovered 14 linkages connecting blood lipids, metabolites, and other cardiometabolic traits. Our study highlights the significance of gut microbiota-derived metabolites in lipid metabolism. Most of the identified cross-sectional associations may be due to the lipids having a predicted causal effect on metabolites, but not vice versa, nor are they due to family confounding. These findings shed new light on lipid metabolism and personalized management of cardiometabolic diseases.

Dyslipidemia is a major risk factor for cardiometabolic diseases (CMDs) (1, 2) and a leading driver of global morbidity and mortality (3, 4). Several genetic and environmental factors have been found to potentially influence blood lipids (5, 6). In recent years, the gut microbiota has emerged as a virtual endocrine organ that influences human health and disease (7), with its effects mediated in part through its by-products, such as metabolites (8). Specifically, gut microbiota-derived metabolites, such as branched-chain amino acids (BCAAs) and bile acids (BAs), have been implicated in the development of metabolic disorders, cardiovascular diseases, and lifespan (9, 10, 11). Among them, many metabolites have already been identified to be associated with lipid levels in animal and epidemiological studies (12, 13, 14). However, current studies have reported mixed evidence, which may be due to differences in study design, sample size, and adjustment for confounders like genetic background (15, 16). Most reported metabolite-lipid associations have come from cross-sectional designs. The causal nature of the association, in other words, whether the metabolite has a possible causal effect on blood lipids or vice versa, remains unclear. The cross-sectional associations identified may also be due to family confounders. The Inference about Causation through Examination of FAmiliaL CONfounding (ICE FALCON) analysis, a novel family-based analytical approach using cross-sectional data (in particular twin data), provides an opportunity to explore predicted causal relationships between lipids and metabolites (17). ICE FALCON could provide the same causal evidence as Mendelian randomization (MR) analysis; furthermore, it differs in that, using the nature of twins, it takes into account all familial causes of exposure and outcome rather than limited factors just captured by measured genetic variants (18).

As one of the cardiometabolic traits, blood lipid is closely related to other cardiometabolic traits, like glycemic traits, including blood glucose (GLU), glycated hemoglobin A1c (HbA1c), and homeostatic model assessment for insulin resistance (HOMA-IR), and blood pressure traits, including systolic blood pressure (SBP) and diastolic blood pressure (DBP). Elucidating the role of metabolites in the interconnection of cardiometabolic traits may help untangle mechanisms underlying downstream clinical diseases, particularly cardiovascular disease and type 2 diabetes mellitus (T2DM).

In this study, we conducted a targeted metabolomics study that covered a wide range of gut microbiota-derived metabolites and metabolites in core metabolic pathways in monozygotic (MZ) twins from the Chinese National Twin Registry (CNTR) to address 3 aims: (1) to reveal the metabolic signature of lipid parameters; (2) to investigate the predicted causal associations between metabolites and lipid parameters; and (3) to identify the role of metabolites between blood lipids and other cardiometabolic traits.

## Material and Methods

### Study participants

The current study was based on CNTR, the largest national twin registry in China, which has recruited 61,566 twin pairs since 2001. Details on the study design of CNTR have been described previously (19). We selected participants who underwent at least one detailed investigation, including an interview-based questionnaire, a physical examination and a blood biochemical test, during the follow-up surveys in 2013 or 2018, and 457 MZ twins were included in the targeted metabolomic analysis of serum samples. Participants were excluded if they were pregnant, had unclear serum lipid status, or were taking lipid-lowering medications. In addition, we performed principal component analysis based on targeted metabolomics data to detect and remove sample outliers. In total, 437 MZ twins (210 twin pairs and 17 unrelated individuals) were included in our study.

### Measurement of epidemiological characteristics, blood lipids, and other cardiometabolic traits

Demographic characteristics and medication history were collected via a uniform interview-based questionnaire. The zygosity of twins was determined based on the method of the peas-in-a-pod questionnaire, which was reported to be 86.98% accurate compared to DNA genotyping (20). Twins who looked like two peas in a pod or were mixed up by strangers, as well as having the same age and gender, were considered MZ twins, while those who looked completely different, were not mixed up, or were of different genders were defined as dizygotic twins.

The study collected blood lipid parameters, including triglyceride (TG), total cholesterol (TC), low-density lipoprotein-cholesterol (LDL-C) and high-density lipoprotein-cholesterol (HDL-C), and seven cardiometabolic traits, including GLU, HbA1c, HOMA-IR, SBP, DBP, blood uric acid and high-sensitivity C reactive protein (hsCRP). Blood samples for blood biochemical tests were drawn from participants after a 12-h fast. More details are described in supplemental File S1.

### Targeted metabolomics

The targeted metabolomics approach was based on measurements with a high throughput targeted quantification for metabolites kit (HM350 Metabolome) performed at Beijing Genomic Institute (BGI, Shenzhen China), using LC-MS/MS. This validated targeted assay allows for the simultaneous detection and quantification of 350 metabolites in serum samples, covering a wide range of important metabolites and core metabolic pathways, of which more than 220 metabolites are related to the gut microbiome. Metabolites with an analytical coefficient of variation above 30% in quality control (QC) samples were excluded. In addition, metabolites with occurrence below 50% in QC samples were removed, while for metabolites detected above 50% in QC samples, measurements below the limit of detection were imputed with the lowest measured concentration. Overall, 248 metabolites were detected in the serum samples of the participants, including 15 classes (amino acids, benzenoids, BAs, carbohydrates, carnitines, fatty acids, indoles, organic acids, and so on) and 154 metabolites related to gut microbiome (supplemental Table S1). Details of sample pretreatment, LC-MS/MS detection and quantification and raw data processing, including information on reagents and internal standards, chromatographic conditions and MS parameters, are described in supplemental File S1.

### Statistical analysis

**Fig. 1:**
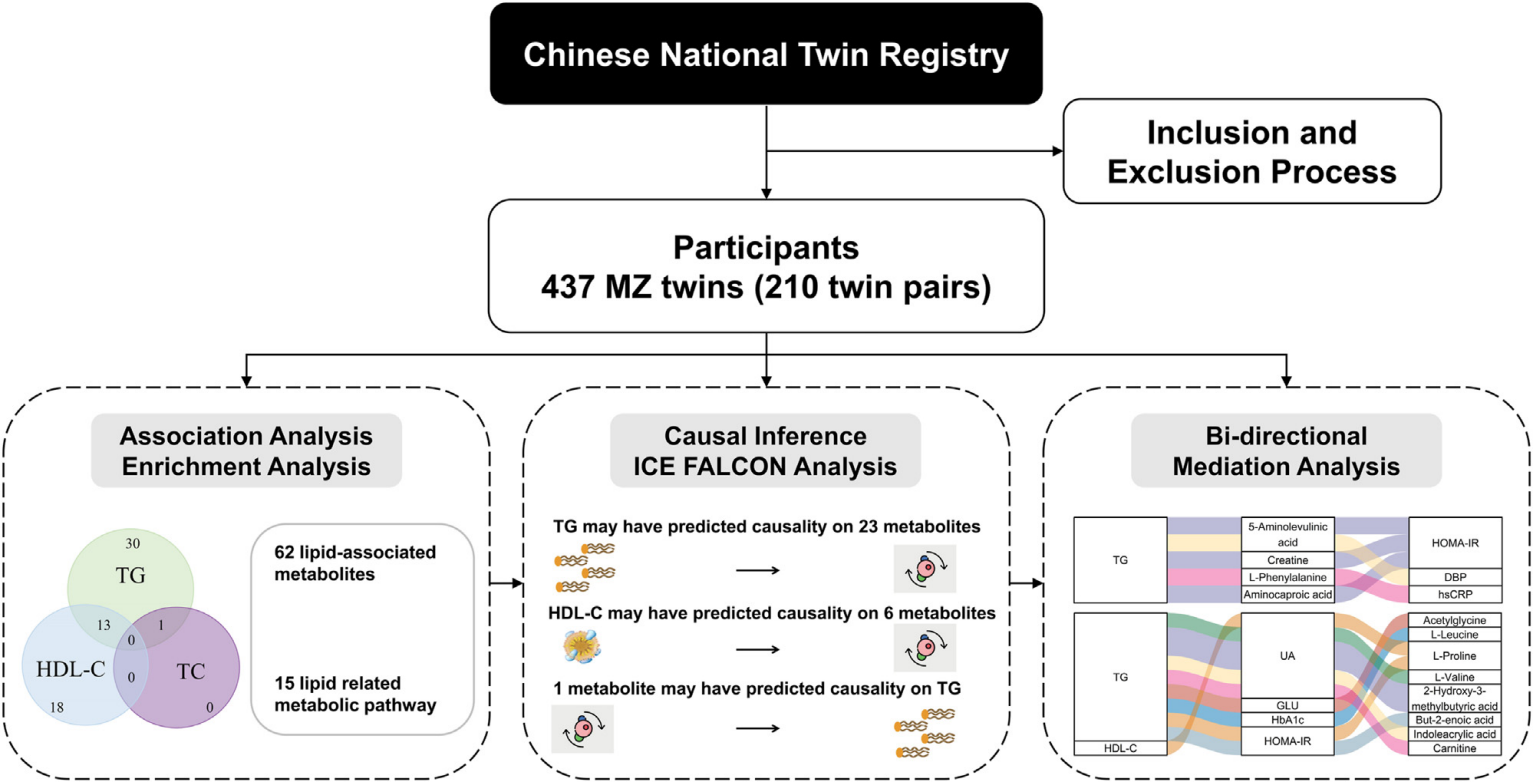
Workflow diagram. Note: DBP: diastolic blood pressure; GLU: blood glucose; HbA1c: glycated hemoglobin A1c; HDL-C: high-density lipoprotein-cholesterol; HOMA-IR: homeostatic model assessment for insulin resistance; hsCRP: high-sensitivity C reactive protein; ICE FALCON: Inference about Causation through Examination of FAmiliaL CONfounding; MZ, monozygotic; TC, total cholesterol; TG, triglyceride; UA, uric acid.

An overview of the analysis procedures is provided in Fig. 1. Each metabolite was first log-transformed and then standardized by subtracting its mean and dividing by its SD. Four lipid parameters and cardiometabolic traits, except SBP and DBP, were log-transformed. All statistical analyses were performed using R version 4.1.2 (https://cran.rstudio.com/bin/windows/base/old/4.1.2/).

#### Associations between metabolites and four lipid parameters

To account for the family relationships, mixed-effect linear regression models were fitted to assess the associations between each metabolite and four lipid parameters using the R package *lme4*, adjusting for age, gender (male vs. female), region (northern China vs. southern China) and follow-up survey year (2013 vs. 2018) as fixed effects, and the twin ID, which was unique to each twin pair, as a random effect. The Benjamini-Hochberg procedure was applied to control the false discovery rate (FDR).

#### Pathway analysis

The metabolic pathway enrichment of metabolites associated with lipid parameters was performed using the Kyoto Encyclopedia of Genes and Genomes database by MetaboAnalyst 5.0 (21).

#### ICE FALCON analysis

To further explore the causal inferences between metabolites and lipid parameters, we carried out ICE FALCON analysis, which can use observational data from related individuals, especially twin data, to investigate whether the association is due to familial confounding factors or possible causal effects through statistical models (17). Current studies have found that compared to MR, ICE FALCON could provide similar causal evidence and does not rely on strong assumptions (17, 18). ICE FALCON analysis is a novel regression modeling approach introduced by Dite *et al.* (22) in 2008. In recent years, this method has been combined with concepts from original path analysis proposed by Sewall Wright (23) and directed acyclic graphs commonly used in epidemiology (24), decomposing the correlation between traits across twins into different parts, including confounding effects and causal effects from diverse pathways, as illustrated in supplemental Fig. S1 and supplemental File S1.

In this study, data from 420 MZ individuals (210 twin pairs) were used for ICE FALCON analysis. We fitted three generalized estimating equations models, first assuming each metabolite to be X (predictor variable) and each lipid parameter to be Y (outcome variable), and then reversing X and Y to undertake the same analyses. To estimate changes in regression coefficients and standard errors, we used a one-sided test and a nonparametric bootstrap method that randomly sampled twin pairs with replacement to produce 1,000 new datasets, each with the same sample size as the original dataset. For each dataset, ICE FALCON was applied to compute changes in regression coefficients. Thus, the SD of 1,000 bootstraps was used as the SE. More information on ICE FALCON analysis can be found in supplemental File S1.

#### MR analysis

For further validation of our finding, we conducted the two-sample bidirectional MR to explore the possible causality between lipid parameters and metabolites identified in our ICE FALCON analysis. Genetic variants employed as instrumental variables for MR analysis were obtained from publicly available genome-wide association study (GWAS) summary statistics, with lipid-associated SNPs taken from a study by Richardson *et al.* (25), and metabolite-associated SNPs taken from studies by Shin *et al.* (26), Richardson *et al.* (27), Panyard *et al.* (28), and Yin *et al.* (29), found in the OpenGWAS database (30). SNPs were clumped at *r*^2^ < 0.001 and 10, 000 kb. MR analyses were performed with the R package TwoSampleMR (github.com/MRCIEU/TwoSampleMR). Considering that there were insufficient genetic data for most metabolites in our study, we only conducted MR analyses for a few metabolites and lipid parameters. We used the inverse variance-weighting (IVW) method for the primary MR analyses, as well as MR-Egger and weighted median methods for sensitivity analyses. We performed the analyses in both directions. Since the MR analysis is confirmatory, a nominal *P*-value < 0.05 was used as the significance threshold. More information on MR analysis can be found in supplemental File S1.

#### Mediation analysis

Based on the direction of the association between metabolites and lipids obtained in ICE FALCON analysis, we inferred potential pathways between metabolites, blood lipids, and seven cardiometabolic traits through bidirectional mediation analysis (31). Metabolites in mediation analysis were required to be associated with cardiometabolic traits using Pearson correlation (*P* < 0.05). Mediation models were adjusted for age, gender, region, and follow-up survey year. Mediation analyses were conducted using the R package mediation.

#### Sensitivity analysis

To test the robustness of our findings, we performed two sensitivity analyses. First, we additionally adjusted for body mass index (BMI) when fitting mixed-effects linear regression models to assess the association between each metabolite and the four lipid parameters. Second, according to the BMI cutoff point of 24 kg/m^2^ recommended for defining overweight in China (32), we conducted overweight-stratified analyses (BMI<24 vs. BMI≥24) to see if the association between metabolites and lipid parameters varied based on the overweight status. Statistical significance of the interaction effects was assessed by comparing models with and without the interaction term via the likelihood ratio test.

### Ethics approval and consent to participate

The study protocol was approved by the Biomedical Ethics Committee at Peking University, Beijing, China (IRB00001052-13022/14021), and all participants provided written informed consent. Our study conforms to the Declaration of Helsinki principles.

## Results

### Characteristics of the participants

A total of 437 participants, including 210 MZ twin pairs, were eligible for our study. Among them, 69.3% were male, and the average age was 52.05 years old. The median concentrations of TG, TC, LDL-C, and HDL-C were 1.46, 4.88, 2.59, and 1.24 mmol/L, respectively. Table 1 describes the detailed demographic information of the participants. The within-pair differences of 248 metabolite levels are shown in supplemental Figs. S2–S4.

**Table 1:** Basic characteristics of the study participants

Characteristic	Overall (n = 437)
Age, mean (SD; years)	52.05 (10.05)
Female, n (%)	134 (30.7%)
Southern region, n (%)	326 (74.6%)
TG, median (IQR; mmol/L)^a^	1.46 (1.05–2.16)
TC, median (IQR; mmol/L)	4.88 (4.22–5.52)
LDL-C, median (IQR; mmol/L)	2.59 (2.10–3.09)
HDL-C, median (IQR; mmol/L)	1.24 (1.04–1.51)
BMI, mean (SD; kg/m^2^)^a^	25.08 (3.34)
GLU, median (IQR; mmol/L)	5.53 (4.98–6.68)
HbA1c, median (IQR; %)^a^	5.70 (5.40–6.30)
HOMA-IR, median (IQR)^a^	1.94 (1.16–3.27)
SBP, mean (SD; mmHg)	142.95 (21.36)
DBP, mean (SD; mmHg)	87.58 (12.48)
UA, median (IQR; μmol/L)	327.25 (269.60–385.00)
hsCRP, median (IQR; mg/L)	0.99 (0.57–1.86)

Note: Quantitative variables were reported as means and standard deviations (SD) or medians and interquartile range (IQR), and categorical variables as numbers and percentages. BMI, body mass index; DBP, diastolic blood pressure; GLU, blood glucose; hsCRP, high-sensitivity C reactive protein; HDL-C, high-density lipoprotein-cholesterol; HbA1c, glycated hemoglobin A1c; HOMA-IR, homeostatic model assessment for insulin resistance; LDL-C, low-density lipoprotein-cholesterol; SBP, systolic blood pressure; TC, total cholesterol; TG, triglyceride; UA, uric acid. ^a^TG: 1 missing value, BMI: 4 missing values; HbA1c: 36 missing values; HOMA-IR: 6 missing values.

### Associations between metabolites and four lipid parameters

**Fig. 2:**
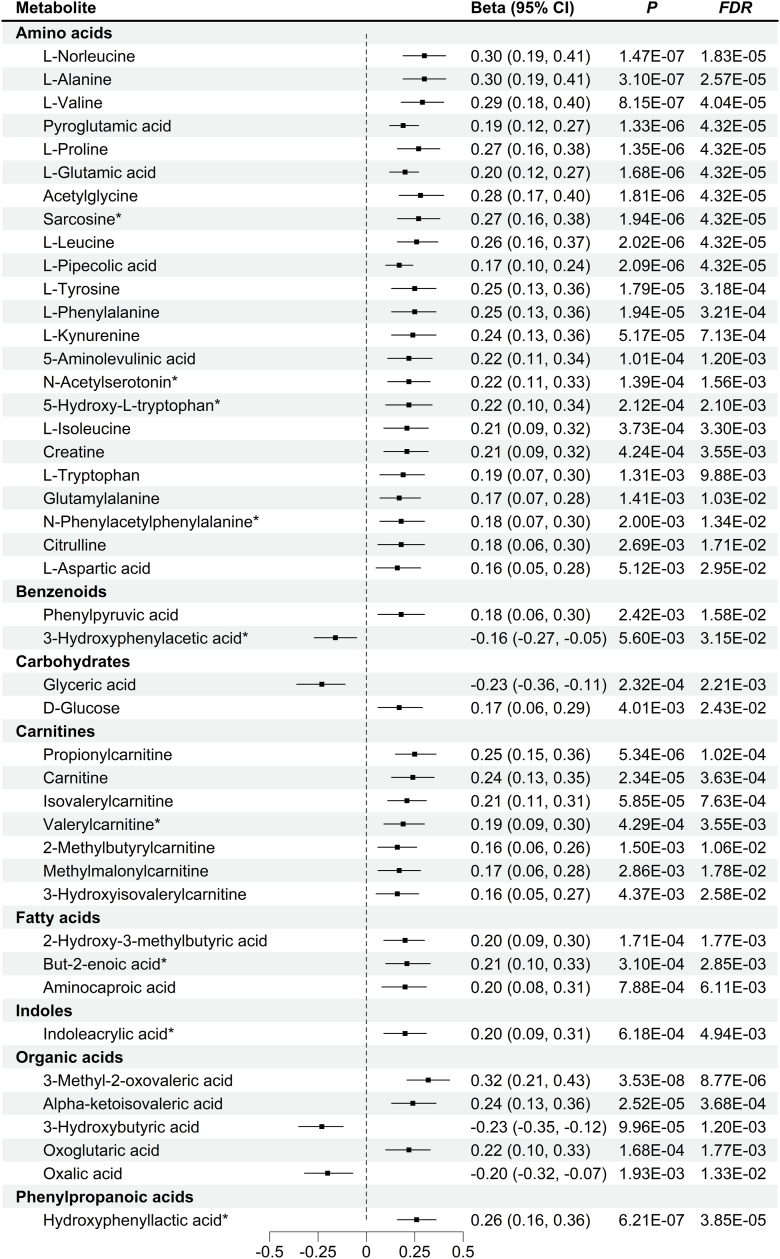
Associations between metabolites and triglyceride. Note: Superscript stars (∗) represent newly identified metabolites associated with triglyceride (TG).

**Fig. 3:**
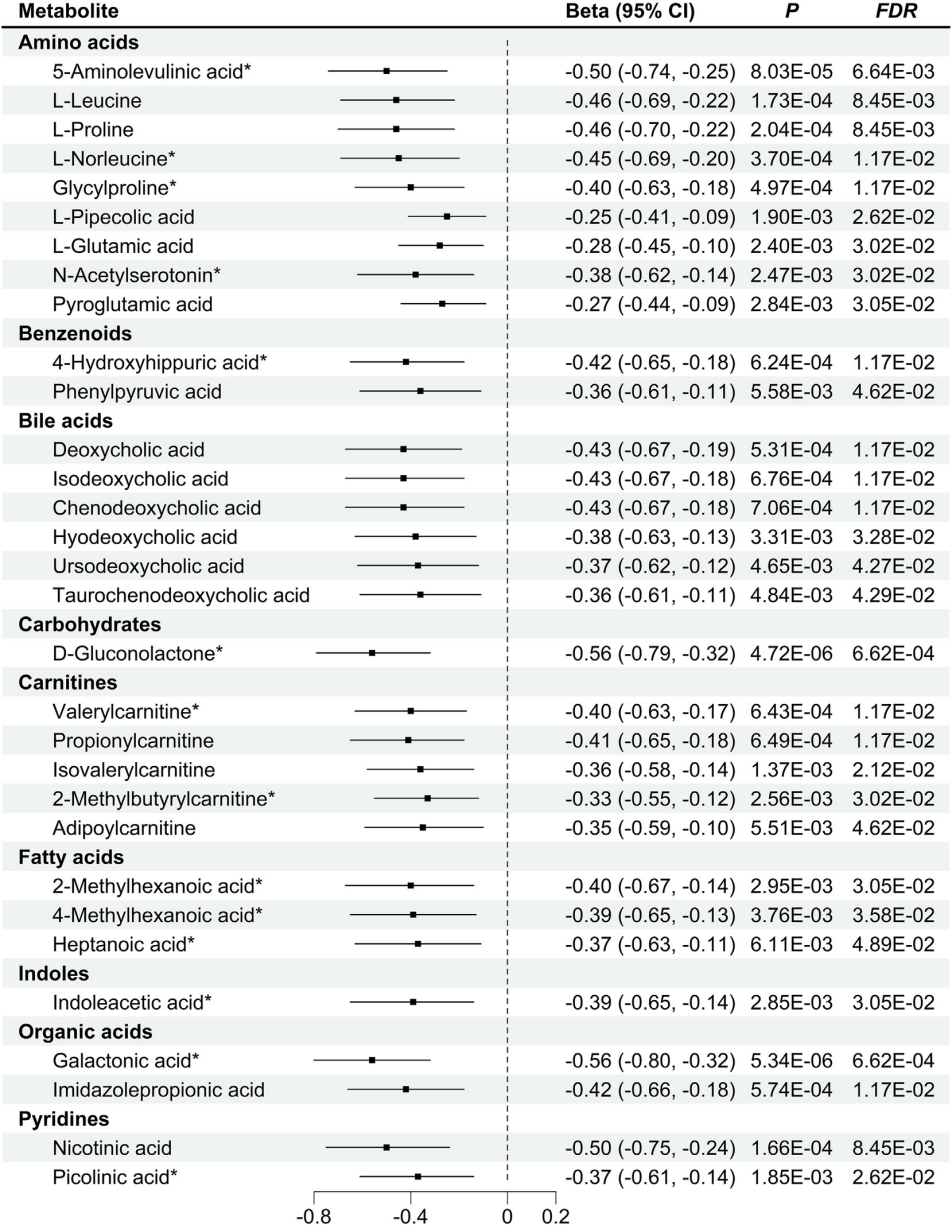
Associations between metabolites and high-density lipoprotein-cholesterol. Note: Superscript stars (∗) represent newly identified metabolites associated with high-density lipoprotein-cholesterol (HDL-C).

Among 248 metabolites, 44, 1, and 31 were associated with TG, TC, and HDL-C, respectively, at FDR <5%, of which 35, 1, and 20 were gut microbiota-derived metabolites. Seven metabolites were associated with LDL-C at uncorrected *P*-value <5%, but there were no significant associations after FDR adjustment (supplemental Table S2). Specifically, 23 amino acids, one benzenoid, one carbohydrate, seven carnitines, three fatty acids, one indole, three organic acids, and one phenylpropanoic acid were positively associated with TG. Conversely, there were negative associations of one benzenoid, one carbohydrate, and two organic acids with TG (Fig. 2). It was observed that one fatty acid was positively associated with TC (supplemental Table S3). In addition, HDL-C was negatively associated with 31 metabolites, including nine amino acids, two benzenoids, six BAs, one carbohydrate, five carnitines, three fatty acids, one indole, two organic acids, and two pyridines (Fig. 3). Interestingly, we found an overlap between TC-associated and TG-associated metabolites, which was but-2 enoic acid. There were also 13 overlapping metabolites between HDL-C and TG, which included eight amino acids (L-norleucine, pyroglutamic acid, L-proline, L-glutamic acid, L-leucine, L-pipecolic acid, 5-aminolevulinic acid, and N-acetylserotonin), one benzenoid (phenylpyruvic acid), and four carnitines (propionylcarnitine, isovalerylcarnitine, valerylcarnitine, and 2-methylbutyrylcarnitine). In the sensitivity analyses, results were largely unchanged after additional adjustment for BMI (supplemental Table S4). The stratified analysis showed that most of the associations between metabolites and lipid parameters did not significant differ by overweight status. (*P* _interaction_>0.05, supplemental Tables S5 and S6).

### Pathway analysis

The result of pathway analysis is shown in supplemental Table S7. The metabolic pathway common to TG and HDL-C was mainly the amino acid metabolism pathway, including phenylalanine metabolism, D-glutamine and D-glutamate metabolism, alanine, aspartate and glutamate metabolism, arginine and proline metabolism, and arginine biosynthesis. There were also differences: the pathway involving BCAAs, like valine, leucine, and isoleucine degradation was unique to TG, while the primary BA biosynthesis pathway was unique to HDL-C.

### Causal relationships between metabolites and lipid parameters

Table 2 exhibits the ICE FALCON results for lipid parameters (TG and HDL-C) and the top five metabolites with the smallest FDR values in the mixed-effect linear regression analysis. By exploring the causation underlying the association between metabolites and TG, the ICE FALCON results were consistent with TG having a possible causal effect on 23 metabolites involving 14 amino acids, one carbohydrate, one carnitine, three fatty acids, one indole, two organic acids, and one phenylpropanoic acid, and one organic acid having a possible causal effect on TG (supplemental Table S8). Among them, 20 metabolites were related to gut microbiota. Here, we take L-valine as an example. From the analysis where L-valine was the predictor and TG the outcome, a twin’s TG was associated with the twin’s own L-valine level (Model 1; β_self_ = 0.1553, 95% confidence interval (CI): 0.0522, 0.2583), but not with the co-twin’s L-valine level (Model 2; β_co-twin_ = 0.0546, 95% CI: −0.0322, 0.1415). However, in Model 3, after adjusting for the twin’s own L-valine level, there was a positive association between the co-twin’s L-valine level and the twin’s own TG (β′_co-twin_ = 0.1230, 95% CI: 0.0244, 0.2216), and the change between β′_co-twin_ and β_co-twin_ was significant (*P* = 5.73E-05). Consistent with the expectation of supplemental Fig. S1C, the results showed the predicted causal evidence of TG on L-valine. We then reversed the predictor variable and the outcome variable and fitted the same models. In the analysis in which TG was the predictor and L-valine was the outcome, a twin’s L-valine level was positively associated with the twin’s own TG (Model 1; β_self_ = 0.2958, 95% CI: 0.1853, 0.4064) and with the co-twin’s TG (Model 2; β_co-twin_ = 0.1978, 95% CI: 0.0773, 0.3183). Conditioning on the co-twin’s TG (Model 3), β′_self_ remained unchanged compared with β_self_ in Model 1 (*P =* 0.37), while conditioning on the twin’s own TG (Model 3), β′_co-twin_ attenuated to become null (β′_co-twin_ = 0.0853, 95% CI: −0.0609, 0.2320). This attenuation was significant (*P* = 5.52E-03). The results were consistent with the expectation of supplemental Fig. S1B rather than supplemental Fig. S1C, indicating that TG may have a causal effect on L-valine, but not in the opposite direction.

**Table 2:** Results from the ICE FALCON analysis for metabolites and blood lipids

Metabolite	Class	Coefficient	Metabolite to Lipid Parameters				Lipid Parameters to Metabolite			
			Model 1	Model 2	Model 3	Change	Model 1	Model 2	Model 3	Change
			Estimate (*P*)	Estimate (*P*)	Estimate (*P*)	Estimate (*P*)	Estimate (*P*)	Estimate (*P*)	Estimate (*P*)	Estimate (*P*)
Triglyceride										
3-Methyl-2-oxovaleric acid	Organic acids	βself	0.1832 (2.95E-07)	-	0.2307 (7.47E-10)	0.0475 (1.04E-07)	0.3416 (8.70E-11)	-	0.3290 (2.56E-08)	−0.0127 (7.14E-01)
		βco-twin	-	0.0165 (6.48E-01)	0.1162 (1.88E-03)	0.0997 (1.07E-11)	-	0.1918 (9.26E-04)	0.0265 (6.62E-01)	−0.1653 (1.17E-05)
L-Norleucine	Amino acids	βself	0.1783 (5.67E-06)	-	0.2200 (1.03E-08)	0.0417 (1.61E-07)	0.3206 (3.39E-09)	-	0.3123 (7.66E-07)	−0.0083 (8.15E-01)
		βco-twin	-	0.0115 (7.53E-01)	0.1042 (5.41E-03)	0.0927 (5.37E-12)	-	0.1681 (2.37E-03)	0.0181 (7.69E-01)	−0.1500 (2.04E-04)
L-Alanine	Amino acids	βself	0.1456 (9.00E-05)	-	0.1892 (9.84E-06)	0.0437 (1.08E-15)	0.2742 (1.90E-07)	-	0.2862 (2.20E-05)	0.0120 (7.34E-01)
		βco-twin	-	−0.0123 (7.21E-01)	0.0864 (2.11E-02)	0.0987 (5.25E-23)	-	0.1346 (1.31E-02)	−0.0225 (7.35E-01)	−0.1571 (5.50E-05)
Hydroxyphenyllactic acid	Phenylpropanoic acids	βself	0.1723 (3.91E-05)	-	0.2103 (1.36E-06)	0.0379 (6.43E-04)	0.2555 (9.08E-08)	-	0.2337 (1.45E-05)	−0.0218 (5.19E-01)
		βco-twin	-	0.0437 (2.99E-01)	0.1154 (7.80E-03)	0.0717 (2.76E-07)	-	0.1506 (2.89E-03)	0.0551 (3.19E-01)	−0.0956 (4.61E-03)
L-Valine	Amino acids	βself	0.1553 (3.14E-03)	-	0.1966 (7.38E-05)	0.0414 (4.61E-04)	0.2958 (1.56E-07)	-	0.2600 (2.69E-04)	−0.0358 (3.73E-01)
		βco-twin	-	0.0546 (2.18E-01)	0.1230 (1.44E-02)	0.0684 (5.73E-05)	-	0.1978 (1.29E-03)	0.0853 (2.53E-01)	−0.1125 (5.52E-03)
High-density lipoprotein-cholesterol										
D-Gluconolactone	Carbohydrates	βself	−0.0374 (4.46E-03)	-	−0.0692 (7.58E-05)	−0.0318 (5.06E-27)	−0.4266 (1.83E-04)	-	−0.4364 (1.08E-02)	−0.0098 (9.29E-01)
		βco-twin	-	0.0012 (9.32E-01)	−0.0465 (1.36E-02)	−0.0477 (5.52E-28)	-	−0.2938 (8.02E-03)	0.0140 (9.32E-01)	0.3078 (8.25E-03)
Galactonic acid	Organic acids	βself	−0.0371 (4.08E-03)	-	−0.0708 (3.32E-05)	−0.0337 (4.76E-34)	−0.4442 (8.50E-05)	-	−0.4419 (8.91E-03)	0.0024 (9.82E-01)
		βco-twin	-	−0.0003 (9.83E-01)	−0.0492 (5.96E-03)	−0.0489 (1.43E-36)	-	−0.3164 (3.46E-03)	−0.0034 (9.83E-01)	0.3130 (4.67E-03)
5-Aminolevulinic acid	Amino acids	βself	−0.0533 (6.75E-04)	-	−0.0783 (1.02E-04)	−0.0250 (1.51E-10)	−0.5417 (1.28E-05)	-	−0.5783 (3.29E-04)	−0.0366 (7.31E-01)
		βco-twin	-	0.0057 (7.00E-01)	−0.0420 (2.36E-02)	−0.0477 (2.33E-19)	-	−0.3051 (1.12E-02)	0.0591 (6.93E-01)	0.3642 (3.14E-03)
Nicotinic acid	Pyridines	βself	−0.0438 (4.96E-03)	-	−0.0642 (2.43E-03)	−0.0204 (5.93E-50)	−0.4689 (2.02E-03)	-	−0.5987 (7.45E-03)	−0.1299 (2.87E-01)
		βco-twin	-	0.0144 (2.72E-01)	−0.0299 (9.20E-02)	−0.0443 (3.42E-61)	-	−0.2267 (5.78E-02)	0.1893 (2.71E-01)	0.4160 (9.17E-03)
L-Leucine	Amino acids	βself	−0.0599 (5.93E-04)		−0.0829 (8.07E-07)	−0.0230 (1.75E-07)	−0.5134 (1.61E-06)		−0.6010 (4.79E-04)	−0.0876 (5.75E-01)
		βco-twin		0.0147 (5.11E-01)	−0.0374 (9.42E-02)	−0.0521 (4.47E-21)		−0.2446 (9.85E-02)	0.1398 (5.05E-01)	0.3844 (1.82E-03)

*Note*: In Model 1, the formula is E(Y_self_) = α+β_self_X_self_. The “Estimate (*P*)” of Model 1 is the estimate and *P* value of β_self_.

In Model 2, the formula is E(Y_self_) = α+β_co-twin_X_co-twin_. The “Estimate (*P*)” of Model 2 is the estimate and *P* value of β_co-twin_.

In Model 3, the formula is E(Y_self_) = α+β′_self_X_self_+β′_co-twin_X_co-twin_. The “Estimate (*P*)” in the upper line refers to the estimate and *P* value of β′_self_, while in the lower line, it refers to the estimate and *P* value of β′_co-twin_.

The “Estimate (*P*)” of the “Change” in the upper line means the difference between β_self_ (Model 1) and β′_self_ (Model 3), while in the lower line, it refers to the difference between β_co-twin_ (Model 2) and β′_co-twin_ (Model 3).

Meanwhile, the ICE FALCON results were consistent with HDL-C having a possible causal effect on metabolites, specifically, D-gluconolactone, galactonic acid, 5-aminolevulinic acid, L-proline, L-norleucine, and 4-hydroxyhippuric acid, two of which were gut microbiota-derived metabolites (supplemental Table S9). In addition, it was found that both TG and HDL-C may have a possible causal effect on 5-aminolevulinic acid, L-proline, and L-norleucine. However, ICE FALCON analysis indicated no predicted causal relationship between but-2-enoic acid and TC (supplemental Table S10).

In the two-sample MR analysis, the standard IVW analysis showed that genetically estimated higher TG levels were significantly associated with elevated L-alanine, L-tyrosine, L-tryptophan, and 3-methyl-2-oxovaleric acid levels. Although the significance differed between methods, the estimated causal effects consistently showed similar magnitude and direction across IVW, MR-Egger, and weighted median. Limited evidence of horizontal pleiotropy was detected using the MR-Egger intercept test for these MR effects. However, the heterogeneity for most of these MR effects was high based on Cochran’s Q-test, indicating the complexity in causality (supplemental Table S11).

In summary, it is important to note that statistically significant ICE FALCON and MR results are insufficient to claim a causality between trait pairs, but only provide suggestive evidence for further investigation. Therefore, these possible causal relationships identified in this study should be interpreted with caution.

### Mediation analysis identified linkages between blood lipids, metabolites, and other cardiometabolic traits

**Fig. 4:**
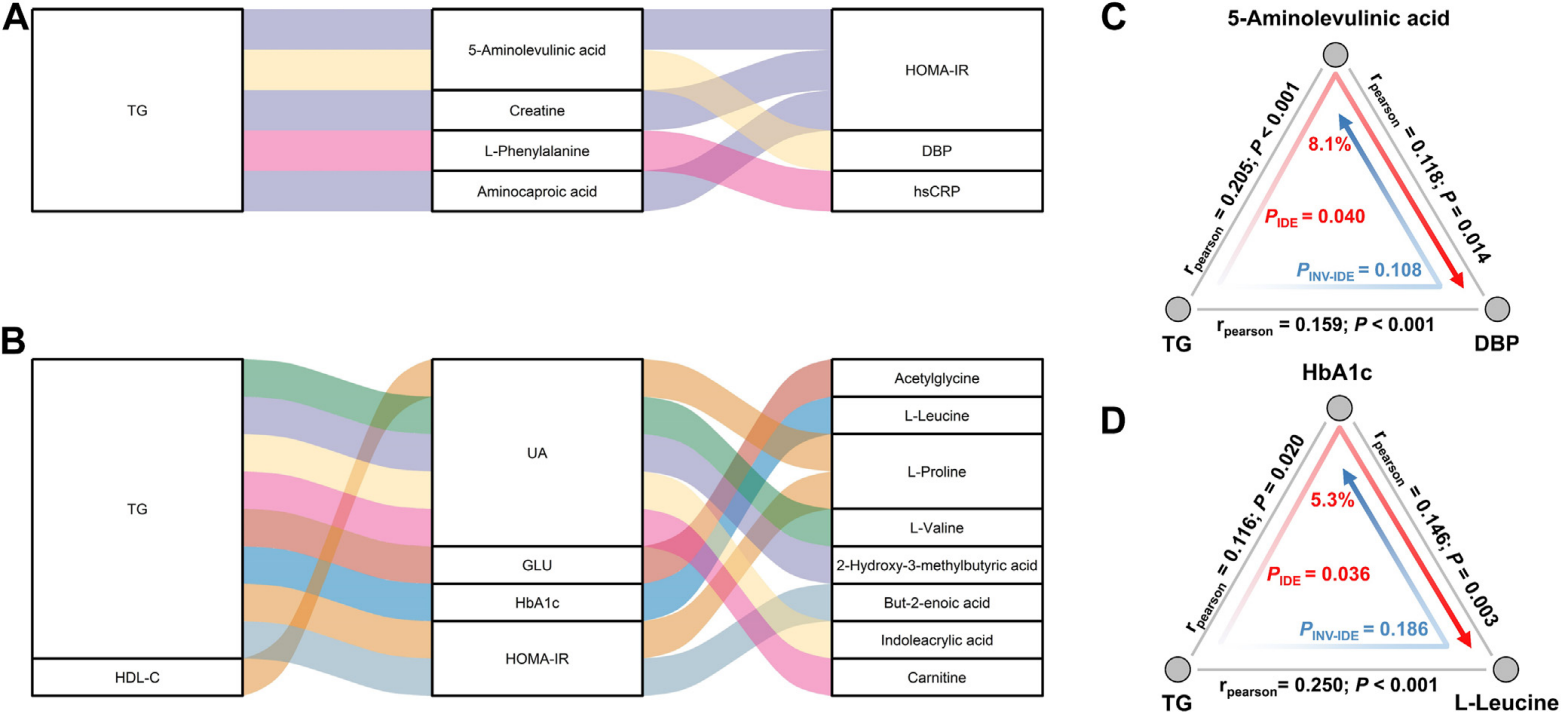
Mediation linkages among blood lipids, metabolites, and other cardiometabolic traits. A: The parallel coordinates chart shows the five significant mediation effects of metabolites. The left panel shows the blood lipids, the middle panel shows the metabolites, and the right panel shows the cardiometabolic traits. The curved lines across the panels indicate the mediation effects, while the colors correspond to different traits. B: The parallel coordinates chart shows the nine significant mediation effects of cardiometabolic traits. The left panel shows the blood lipids, the middle panel shows the cardiometabolic traits, and the right panel shows the metabolites. The curved lines across the panels indicate the mediation effects, while the colors correspond to different metabolites. C: TG causally contributed to DBP through 5-aminolevulinic acid. D: TG causally contributed to L-leucine through HbA1c. Note: DBP, diastolic blood pressure; GLU, blood glucose; hsCRP, high-sensitivity C reactive protein; HbA1c, glycated hemoglobin A1c; HDL-C, high-density lipoprotein-cholesterol; HOMA-IR, homeostatic model assessment for insulin resistance; TG, triglyceride; UA, uric acid.

Meanwhile, to evaluate whether metabolites can mediate the effects of lipid parameters on other cardiometabolic traits (supplemental Table S12), we applied bidirectional mediation analysis, and identified 14 mediation linkages (*P*_mediation_ < 0.05 and *P*_inverse mediation_ > 0.05, Fig. 4 and supplemental Table S13). Specifically, there were five for TG impact on cardiometabolic traits through metabolites, eight for TG, and one for HDL-C impact on metabolites through cardiometabolic traits. For five linkages that the metabolite can meditate TG impact on cardiometabolic traits, most of them were related to HOMA-IR. It was found that 5-aminolevulinic acid (4%, *P*_mediation_ = 0.034), creatine (4%, *P*_mediation_ = 0.036) and aminocaproic acid (4%, *P*_mediation_ = 0.046) can mediate the effect of TG on HOMA-IR. Surprisingly, 5-aminolevulinic acid also mediated the effect of TG on DBP (8%, *P*_mediation_ = 0.040). In addition, we also observed TG might contribute to the inflammatory responses by affecting the L-phenylalanine level (7%, *P*_mediation_ = 0.036), as seen in hsCRP. On the other hand, for the effects of lipid parameters on metabolites through cardiometabolic traits, uric acid mediated the effect of TG on four metabolites, namely L-valine (13%, *P*_mediation_ = 0.034), carnitine (15%, *P*_mediation_ = 0.034), 2-hydroxy-3-methylbutyric acid (17%, *P*_mediation_ = 0.034) and indoleacrylic acid (19%, *P*_mediation_ = 0.034), and also mediated the effect of HDL-C on L-proline (9%, *P*_mediation_ = 0.032). Besides, our mediation analysis suggested TG may impact on acetylglycine (9%, *P*_mediation_ = 0.034), L-leucine (5%, *P*_mediation_ = 0.036), L-proline (17%, *P*_mediation_ = 0.038), but-2-enoic acid (25%, *P*_mediation_ = 0.026) through glycemic traits including GLU, HbA1c, and HOMA-IR.

## Discussion

In general, we conducted a large targeted metabolomics study in Chinese twins to provide detailed information on the association between metabolites and blood lipids. We discovered 44, 1, and 31 metabolites associated with TG, TC, and HDL-C, most of which were gut microbiota-derived metabolites. Using a novel family-based analytical method, ICE FALCON analysis, we found evidence consistent with TG and HDL-C having a predicted causal effect on 23 and 6 metabolites respectively, and another metabolite having a predicted causal effect on TG. Through bidirectional mediation, 14 mediation linkages were identified, providing insight for revealing the potential regulatory mechanisms between blood lipids, metabolites, and cardiometabolic traits.

Of the metabolites we identified that were associated with blood lipids, many metabolites have been demonstrated in previous epidemiological or animal studies (12, 13, 14), including BCAAs and aromatic amino acids which are currently considered to be the strongest CMD biomarkers, as well as the emerging CMD biomarkers carnitine and BAs. In addition, we newly discovered 9, 1, and 14 metabolites associated with TG, TC, and HDL-C, respectively, some of which have been reported to be related to obesity, T2DM, and cardiovascular disease (33, 34). Although our study reports the first association of these metabolites with lipids, previous studies investigating their biological functions may provide some insights into these associations. For instance, 3-hydroxyphenylacetic acid, a flavonoid metabolite formed by the human gut microbiota, has been found to be vasoactive and lower blood pressure (33). Additionally, changes in 3-hydroxyphenylacetic acid have been reported to be affected by green tea or red wine intake (35, 36) which was observed to be associated with lipid traits (37), thus these metabolites may mediate the green tea-lipid or red wine-lipid relations. Notably, many lipid-associated metabolites are gut microbiota-derived metabolites, which highlights the essential role that gut microbiota may play in the development of lipid metabolism by regulating the levels of bioactive metabolites in the blood. Our study observed overlap in metabolites associated with TG and HDL-C, which were mainly involved in the amino acid metabolism pathway; however, there were also distinctions. One such difference is that BAs were associated with HDL-C levels, whereas they did not show any association with TG. Previous studies have specifically shown that the gut microbiota can play a role in altering the composition of blood lipids, particularly cholesterol, through BA metabolism and microbial products (38, 39). Of note, we did not identify any metabolites significantly associated with LDL-C, despite high LDL-C being a well-known independent cardiometabolic risk factor (40). The reason may be that the twins who participated in our study were relatively healthy. Similar results were obtained in a twin study in which no metabolites were significantly associated with LDL-C (41).

Moreover, our study filled the gap in predicted causality between blood lipids and metabolites. Our ICE FALCON analysis showed evidence consistent with a predicted causal effect of TG and HDL-C on 23 and 6 metabolites, respectively, and another metabolite with predicted causality for TG. Importantly, these predicted causal relationships found were independent of familial confounding factors, such as shared genetic backgrounds and environments, which may also be partially supported by the observation that associations between specific metabolites and blood lipids were consistent across diverse ethnic populations with different genetic and environmental influences (42, 43, 44). From ICE FALCON analysis, for example, we found that TG may have a predicted causal effect on BCAAs, including leucine and valine. BCAAs are essential amino acids and gut microbiota-derived metabolites, which are used to synthesize proteins and also perform in various signaling pathways (45, 46). Previous cohort studies have shown that elevated BCAA levels were associated with the risk of hypertriglyceridemia (16, 47). The available evidence, however, is inconsistent. Interestingly, a bidirectional two-sample MR analysis performed by Doestzada *et al.* (15) was consistent with our findings. The MR analysis revealed potential causal relationships from increased TG levels and reduced HDL-C levels to an increment of BCAA levels, while no significant causal effects of BCAA on TG and HDL-C were detected. Moreover, a great number of causal relationships from lipidomic traits to BCAAs further confirmed that changes in BCAA levels may be a consequence of altered lipid metabolism. The results of another large-scale two-sample MR study conducted by Gagnon *et al.* (8) also did not support a causal impact of BCAAs on TG, but instead supported a null association. The study suggested that the previously reported associations in observational studies may not always be underlying causation, but may be due to biases such as confounding or reverse causality. To further test our findings, we conducted the two-sample bidirectional MR to explore the possible causality between lipids and other metabolites identified in ICE FALCON analyses, and found similar evidence supporting some possible causal relationships. However, the small sample size of the metabolite GWAS could potentially introduce weak instrumental bias (*F* < 10) in the MR analysis, thus leading to inaccurate estimates of causal effects. The MR method requires comprehensive study of the phenotypes of interest and extensive consideration of genetic determinants and confounding factors. In comparison, the ICE FALCON method may have some advantages in providing evidence of causality between trait pairs with the use of the nature of twins (17), particularly when the genetic backgrounds of these traits have not been fully explored. Notably, although we adopted ICE FALCON and MR to imply the possible underlying causal associations, the results were mainly based on observational data and statistical models. Therefore, the interpretation of causality should be made with caution and can only be considered indicative. Future in vitro and in vivo studies are required to validate our findings.

In addition, our bidirectional mediation analysis results observed several mediation linkages that revealed the role of metabolites between blood lipids and cardiometabolic traits. Although the mediation proportions were small, the results were statistically significant, which contained certain theoretical implications for exploring these pathways. Notably, we found HbA1c could mediate the pathway from TG to L-leucine, rather than L-leucine mediating the pathway from TG to HbA1c. Previous studies have shown that leucine can predict future conversion to T2DM (48, 49). However, current research regarding the role of BCAAs in the change of glycemic traits has not been fully established. The MR analysis of Porcu *et al.* (50) not only confirmed the causal role of leucine on T2DM, but also unveiled a strong reverse causal effect of glucose/T2DM on leucine, suggesting that the genetic predisposition to T2DM could trigger early changes in leucine levels before any clinical symptoms occur. Wang *et al.* (51) discovered that insulin resistance (IR), defined as a triad of higher fasting insulin, higher TG, and lower HDL-C, might causally affect levels of circulating BCAAs. Similar to these studies, our finding, a pathway from TG through HbA1c to BCAAs, may shed new light on the metabolic changes between lipid and glycemic profiles or even plausible mechanistic hypotheses for the early onset of T2DM.

The mediation analysis results also filled the gap in potential pathways by which metabolites mediate the effects of TG on cardiometabolic traits. We revealed that TG may influence HOMA-IR through creatine. Creatine is thought to be involved in energy buffering and cellular bioenergetics through the creatine kinase/phosphocreatine system (52), and can be degraded by colonic bacteria and fungi (53). Consistent with our findings, Li *et al.* (44) discovered that abnormal creatine metabolism was associated with hyperlipidemia (HLP). Vangipurapu *et al.* (54) found that creatine significantly reduced insulin sensitivity. Post *et al.* (55) also observed that higher creatine levels were associated with an increased risk of T2DM. In addition, our study revealed that 5-aminolevulinic acid may mediate the effect of TG on HOMA-IR, and also mediate the effect of TG on DBP. When δ-aminolevulate dehydratase is inhibited by lipid disturbances, the enzyme’s substrate 5-aminolevulinic acid may accumulate and further exacerbate oxidative stress (56). Walejko *et al.* (57) reported that a combination of metabolic and microbial characteristics, including elevated 5-aminolevulinic acid levels, was shown in African Americans with hypertension, indicating that higher blood pressure may be associated with higher oxidative stress and inflammation. Besides, L-phenylalanine was also found to mediate the effect of TG on hsCRP. Likewise, Yang *et al.* (42) observed increased levels of phenylacetic acid (a metabolite of phenylalanine) in HLP patients, and suggested that this metabolite may be involved in the potential inflammation caused by HLP.

We acknowledge some limitations of our study. First, the participants in this study were Chinese twins; therefore, all results may not be generalized to other populations. However, previous studies have shown that twins do not systematically differ from the general nontwin population with respect to biomarker profiles and diseases (58). Large-scale multicenter studies or MR studies, including meta-analyses of studies with larger sample sizes, are needed to verify causality. Second, this study did not consider the complex effects of diet, physical activity, and other lifestyle factors on human metabolic pathways. Third, despite conducting targeted metabolomics under optimal conditions, some metabolites, such as important gut microbiota-derived metabolites like trimethylamine N-oxide and betaine, were below the detection limit. Therefore, this study was unable to further explore their relationship with blood lipids.

## Conclusions

In conclusion, our study revealed the metabolic signature of blood lipid parameters and identified 9, 1, and 14 metabolites that showed novel associations with TG, TC, and HDL-C, respectively. We found that most cross-sectional associations identified between metabolites and lipids were due to blood lipids having a predicted causal effect on metabolites, but not vice versa, nor were these relationships due to family confounding. We further identified certain metabolites that may play a role between blood lipids and other cardiometabolic traits. Our findings suggest the significance of gut microbiota-derived metabolites in lipid metabolism and shed new light on a plausible mechanistic hypothesis for CMDs.

## Data Availability

The datasets analyzed during the current study are not publicly available but are available from the corresponding authors on reasonable request.

## Supplemental data

This article contains supplemental data (17, 59).

## Conflict of interest

All the authors declare no conflicts of interest in this study.
