# Supplementary material for: The metabolic signature of blood lipids: a causal inference study using twins
Source: J Lipid Res. 2024 Sep 19;65(9):100625. doi: 10.1016/j.jlr.2024.100625 (PMC11437770; doi:10.1016/j.jlr.2024.100625)
Supplement: Supplemental file [file mmc1.docx]

**Supplemental File 1**

**1 Methods**

**1.1 Measurement of epidemiological characteristics, blood lipids and** **cardiometabolic traits**

Except for the glycated hemoglobin A1c (HbA1c), which was measured in EDTA anticoagulated whole blood, other indicators were measured in serum. The enzymatic colorimetric method was applied to measure triglyceride (TG) and total cholesterol (TC), and the direct approach was utilized for the detection of low-density lipoprotein-cholesterol (LDL-C) and high-density lipoprotein-cholesterol (HDL-C). The blood glucose (GLU) level was detected by a modified hexokinase enzymatic method, HbA1c level was measured by the high-performance liquid chromatography, and chemiluminescence immunoassay on the ADVIA Centaur immunoassay system was used for the insulin level. Insulin resistance was estimated according to homeostasis model assessment (HOMA-IR): HOMA-IR = [fasting glucose (mmol/l) × insulin (U/ml)]/22.5. The serum high-sensitivity C reactive protein (hsCRP) level was measured by immunoturbidometric assay. Systolic blood pressure (SBP) and diastolic blood pressure (DBP) were measured twice in the sitting position after taking a rest of at least 5 min using an OMRON HEM-7200 electronic sphygmomanometer on the right arm. The two sequential measurements were averaged. A third measurement was obtained if the difference between the two measurements was >10 mmHg, and the two closest measurements were averaged. For participants who were using antihypertensive medication, the SBP and DBP levels were adjusted by adding 15 and 10 mmHg, respectively.

**1.2 Targeted metabolomics**

**1.2.1 Sample pretreatment**

120μL pure methanol was added to 20μL thawed serum sample and 20μL internal Standard I, respectively. After vortex at 1200rpm at room temperature for 20 minutes, the sample was centrifuged at 18000g at 4℃ for 20 minutes. Then 30μL supernatant was transferred to a 96-well plate, and was added 20μL derivatization reagent (3-nitrophenylhydrazine) and 20μL EDC working solution in turn. Covered with aluminum film, the 96-well plate was placed in a constant temperature oscillator at 1200rpm at 30℃ for 60 minutes. After that, 100μL precooled 50% methanol was added to each well and mixed at 1200rpm at room temperature for 5 minutes, and then centrifuged at 4000g at 4℃ for 30 minutes. 10μL internal Standard II working solution was added to a new 96-well plate, and then 100μL supernatant were transferred to the96-well plate. After mixing at 650rpm for 5 minutes and sealing the film, samples were prepared for LC-MS/MS analysis.

**1.2.2 LC-MS/MS detection and quantification**

**1.2.2.1 Chromatographic separation**

Waters, ACQUITY UPLC I-Class PLUS System was used to perform the analysis. Waters, BEH C18 column (1.7 μm, 2.1 mm×100 mm) was used. The sampler and column temperature were maintained at 8℃ and 40℃, respectively. The mobile phase consists of (A) 0.1% formic acid with 99.9% water and (B) 30% isopropanol with 70% acetonitrile. The gradient duration program was as follows: 5 minutes hold at 5% B, 4 minutes hold at 70% B, 2 minutes hold at 50% B, 2.5 minutes hold at 22% B, 0.5 minutes hold at 95% B, the above flow rate was maintained at 0.400mL/min; 2 minutes hold at 100% B, the flow rate was at 0.600mL/min; 2 minutes hold at 5% B, the flow rate was at 0.400mL/min.

**1.2.2.2 Mass Spectrometer parameters**

Sciex 6500 QTRAP Plus equipped with an electrospray ionization (ESI) source was used to carry out the mass spectrometric analyses under positive and negative ion modes. The conditions were as follows: The ion source temperature was 550°C. The ion spray voltages were 5500 V and 4500 V in positive ion mode and negative ion mode, respectively. The ion source gas 1, ion source gas 2 and curtain gas, were 60, 60, and 35 psi, respectively. All targets were monitored using multiple reaction-monitoring (MRM) mode.

**1.2.2.3 Raw data processing**

The obtained raw mass spectrometry data was imported into the Skyline software, which generated standard curves based on the specified reference standards and calculated the concentration values of each metabolite in each sample. The method of 7-point correction standard curve was used to quantify metabolites. Data preprocessing involved checking peak-area integration, checking for fit to a standard curve, data validation and correcting plate-to-plate bias, which was described previously(1). A total of 82 quality control (QC) samples were in this study. A QC sample was made by mixing and blending equal volumes from each serum sample, which underwent the same pre-treatment process and mass spectrometry detection process as the samples to be tested. PCA was performed for outlier detection using the preprocessed dataset.

**1.3 Inference about Causation through Examination of FAmiliaL CONfounding (ICE FALCON) analysis**

**1.3.1 Assumption descriptions**

According to the possible directed acyclic graphs (DAGs), if there is a cross-twin cross-trait correlation, that is, the correlation between Y_self_ and X_co-twin_, or between X_self_ and Y_co-twin_, it could be due to (1) familial confounders S_XY_ (Supplemental Figure 1A); (2) X may have a causal effect on Y within an individual, where X_self_ and X_co-twin_ are correlated owing to S_X_ (Supplemental Figure 1B); or (3)Y may have a causal effect on X, where Y_self_ and Y_co-twin_ are correlated owing to S_Y_ (Supplemental Figure 1C). Causality here means that if the predictor variable could be experimentally changed, the expected value of the outcome variable would change(2). Furthermore, it should be noted that individual-specific confounders (C_self_ and C_co-twin_) will not by themselves lead to cross-twin cross-trait correlations.

**1.3.2 Model fitting and expected results**

ICE FALCON analysis will fit three models using generalized estimating equations (GEE) to account for the correlation in Y between twins from the same pair. They are:

Model 1: E(Y_self_)=α+β_self_X_self_

Model 2: E(Y_self_)=α+β_co-twin_X_co-twin_

Model 3: E(Y_self_)=α+β′_self_X_self_+β′_co-twin_X_co-twin_

Under different assumptions, the regression coefficients change differently in the three models. Based on this, ICE FALCON analysis tests which assumption among the above DAGs the data is most consistent with. It is important to note that the ICE FALCON analysis process does not prove any model to be the real representation of nature, but rather indicate whether the data is ‘consistent’ with a specific causal hypothesis. Here is a detailed description of the expected regression results under different assumptions for ICE FALCON analysis:

If the cross-twin cross-trait correlation is only due to familial confounders (supplemental Figure S1A), Y_self_ will be significantly associated with X_self_ (β_self_, Model 1); meanwhile, Y_self_ will also be significantly associated with X_co-twin_ (β_co-twin_, Model 2). With the adjustment of X_self_ in Model 3, there will remain a conditional association between Y_self_ and X_co-twin_ (β′_co-twin_, Model 3), but it is expected to attenuate toward the null compared with β_co-twin_ in Model 2. Likewise, adjusting for X_co-twin_ in Model 3 is expected to attenuate the conditional association between Y_self_ and X_self_ (β′_self_, Model 3) from β_self_ in Model 1 toward the null. Both attenuations will exhibit similar magnitudes.

If the cross-twin cross-trait correlation is only due to a causal effect from X to Y (Supplemental Figure 1B), Y_self_ will be significantly associated with X_self_ (β_self_, Model 1). In Model 2, there are two paths through which Y_self_ and X_co-twin_ are associated: conditioning on the collider Y_co-twin_ and the confounder S_X_ (β_co-twin_, Model 2). In Model 3, after adjusting for X_self_, both paths will be blocked, and β′_co-twin_ will attenuate toward the null. Nevertheless, since conditioning on X_co-twin_ in Model 3 does not affect the path between Y_self_ and X_self_, there still will be an association between them, and β′_self_ will be similar to β_self_ in Model 1.

If the cross-twin cross-trait correlation is only due to a causal effect from Y to X (Supplemental Figure 1C), Y_self_ will be significantly associated with X_self_ (β_self_, Model 1). In Model 2, both paths between Y_self_ and X_co-twin_ are closed. Specifically, the path through X_self_ is blocked owing to X_self_ as a collider, and the path through S_Y_ is blocked since Y_co-twin_ is conditioned on, so β_co-twin_ in Model 2 will be null. In Model 3, after adjusting for X_self_, both paths will be open, and there will be an association between Y_self_ and X_co-twin_ (β′_co-twin_, Model 3).

**1.4 Mendelian randomization (MR) analyses**

For MR analyses, instrumental variables need to meet three assumptions: (1) be strongly associated with the exposure, (2) share no common cause with the outcome, and (3) only affect the outcome through the exposure. To meet the first assumption, for lipid parameters, we selected GWAS *P*<5E-08 and *F*>10 as the thresholds for SNP instrumental variables. For metabolites, we chose a GWAS *P*-value threshold of 5E-08 for SNP instrumental variable selection; if the number of instrumental variables for a metabolite was less than 5 at this threshold, we chose a threshold of 1E-05, all without considering the *F* statistic. To avoid the potential violation of the second MR assumption, for lipid parameters, we removed SNPs that were associated with corresponding metabolites; for metabolites, we removed SNPs that were associated with lipid parameters. To reduce the chance of violating the third assumption, we removed SNPs that were associated with obesity indicators (such as BMI, waist circumference, hip circumference, waist-hip ratio), diabetes indicators (such as blood glucose and HbA1c) and metabolic syndrome.

**Supplemental Figure S1 Possible causal diagrams that explain the cross-twin cross-trait correlation**

**
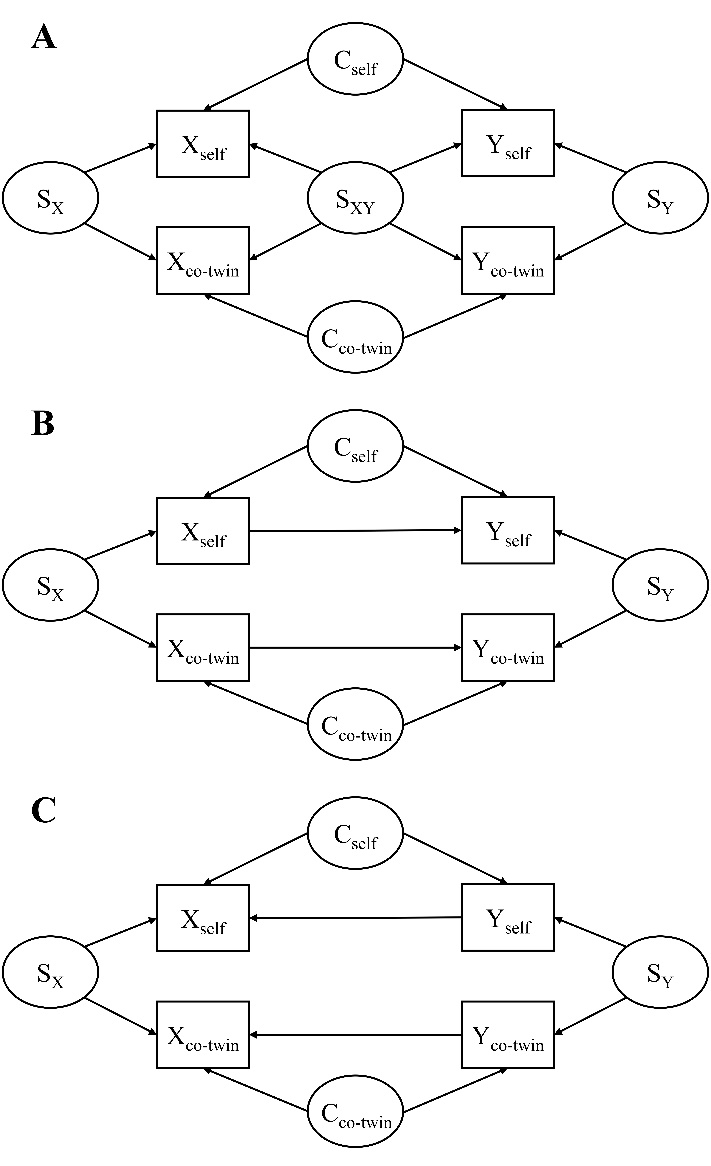
**

*Note*: X: predictor variable; Y: outcome variable; S: unmeasured familial factors, including genetic and non-genetic influences. S_X_: unmeasured familial factors only affect X; S_Y_: unmeasured familial factors only affect Y; S_XY_: unmeasured familial factors affect both X and Y; C: all unmeasured confounders between X and Y that are not shared by twins; self: an individual; co-twin: the individual’s twin. These labels can be interchanged, and both of a pair of twins are included in the analysis. (A). The correlation is due to familial confounding. (B). The correlation is due to the causal effect from X on Y. (C). The correlation is due to the causal effect from Y on X.

**Supplemental Figure S2. Within-pair differences of metabolite levels (64 amino acids)**





**Supplemental Figure S3. Within-pair differences of metabolite levels (20 Benzenoids, 24 Bile acids, 19 Carbohydrates, and 21 Carnitines)**





**Supplemental Figure S4. Within-pair differences of metabolite levels (51 Fatty acids, 27 Organic acids, and 22 in other classes)**





**Reference**

1. Adams, K. J., Pratt, B., Bose, N., Dubois, L. G., St John-Williams, L., Perrott, K. M.*, et al.* (2020) Skyline for Small Molecules: A Unifying Software Package for Quantitative Metabolomics. *J Proteome Res*. 19, 1447-1458.

2. Li, S., Bui, M., Hopper, J. L. (2020) Inference about causation from examination of familial confounding (ICE FALCON): a model for assessing causation analogous to Mendelian randomization. *Int J Epidemiol*. 49, 1259-1269.
